# Extensive Invasion of the Pelvicalyceal System Shortly After Combined Locoregional Treatment of Renal Cell Carcinoma: A Report of an Unusual Complication

**DOI:** 10.7759/cureus.100147

**Published:** 2025-12-26

**Authors:** Hippocrates Moschouris, Chrysovalantis Stylianou, Theodora Adamaki, Dimitra Eirini Evripidi, Konstantinos Arvanitidis

**Affiliations:** 1 Radiology, General Hospital of Piraeus Tzaneio, Athens, GRC; 2 Radiology and Interventional Radiology, Democritus University of Thrace, Alexandroupolis, GRC; 3 Radiology and Interventional Radiology, University General Hospital of Alexandroupolis, Alexandroupolis, GRC

**Keywords:** interventional oncology, interventional radiology complications, kidney sparing, pelvicalyceal system, percutaneous microwave ablation, renal cell carcinoma (rcc), transcatheter arterial embolization

## Abstract

Renal cell carcinoma (RCC) is a relatively common malignancy and is often diagnosed incidentally in imaging in early stages, where interventional radiologic techniques provide competent treatment options with minimal risk for major complications. A 72-year-old woman diagnosed with a 5 cm tumor of her right kidney in computed tomography (CT) with typical findings of RCC opted for percutaneous interventional treatment instead of surgical treatment. Transarterial embolization (TAE), followed by ultrasonographically guided microwave ablation (MWA), was performed with no immediate post-interventional complications. The patient returned 10 weeks post MWA with right flank pain and hematuria, and CT revealed almost complete necrosis at the original tumor site but also significant tumor extension in the upper calyces and pelvis of the right kidney. This was histologically confirmed after nephroureterectomy. The complication is thought to be unrelated to the TAE, and cell implantation potentially occurred due to calyceal penetration during antenna placements. TAE and thermal ablation are established techniques for managing locoregional disease. Urinary tract invasion by treatment-naive RCC has been reported in the literature; however, invasion of the pelvicalyceal system by RCC as a result of ablation should be considered an extremely unusual, unexpected, and severe complication, which impairs the prognosis.

## Introduction

Renal cell carcinoma (RCC) is the most common malignancy of the kidneys and the 14th most common malignancy worldwide [1]. Clinical presentation typically involves the triad of flank pain, macroscopic hematuria, and palpable abdominal mass. However, extensive use of and advances in medical imaging allow the incidental detection of renal lesions in asymptomatic patients and in early stages, without locoregional spread. In such cases, surgical approaches, such as partial or radical nephrectomy, represent the “gold standard” treatment [1]; nevertheless, percutaneous interventional radiologic (IR) approaches (in the form of radiofrequency-, microwave- or cryoablation) offer an effective alternative, particularly for patients with high surgical risk or other contraindications [2,3]. The aforementioned ablative techniques (occasionally combined with transarterial embolization) are not only comparable to surgery in terms of clinical efficacy, but they are also associated with a very favorable safety profile, with less than 5% prevalence of major (mainly hemorrhagic) complications [3]. In this report, a quite unusual and unexpected, non-hemorrhagic event is described, which severely complicated an apparently straightforward attempt for IR management of a solitary RCC.

## Case presentation

**Figure 1:**
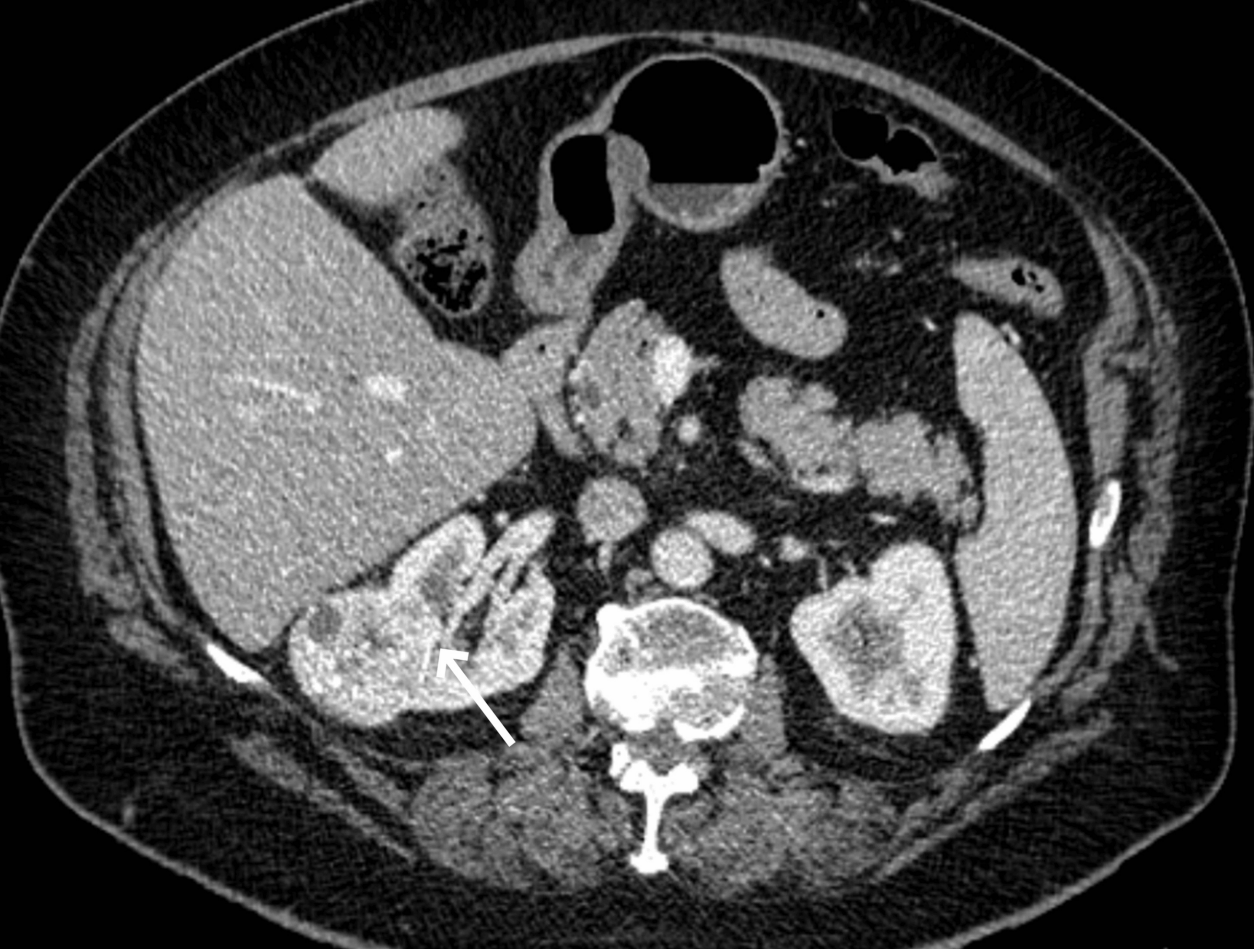
Contrast-enhanced, axial CT image. A hypervascular, partially exophytic right renal tumor. The deepest part of the tumor (arrow) is abutting the renal pelvis.

A right renal tumor measuring 4 x 4.2 x 5 cm was incidentally diagnosed on a contrast-enhanced computed tomography (CECT) scan of a 72-year-old Caucasian woman. Imaging features were typical of RCC (Figure 1). Moreover, further review of the CECT scan excluded distal metastases, locoregional lymphadenopathy, or renal vein/inferior vena cava invasion, thus indicating a T1b N0 M0 (stage 1) RCC. The rest of her medical history was unremarkable, and laboratory tests, including those for renal function, were within normal limits.

**Figure 2:**
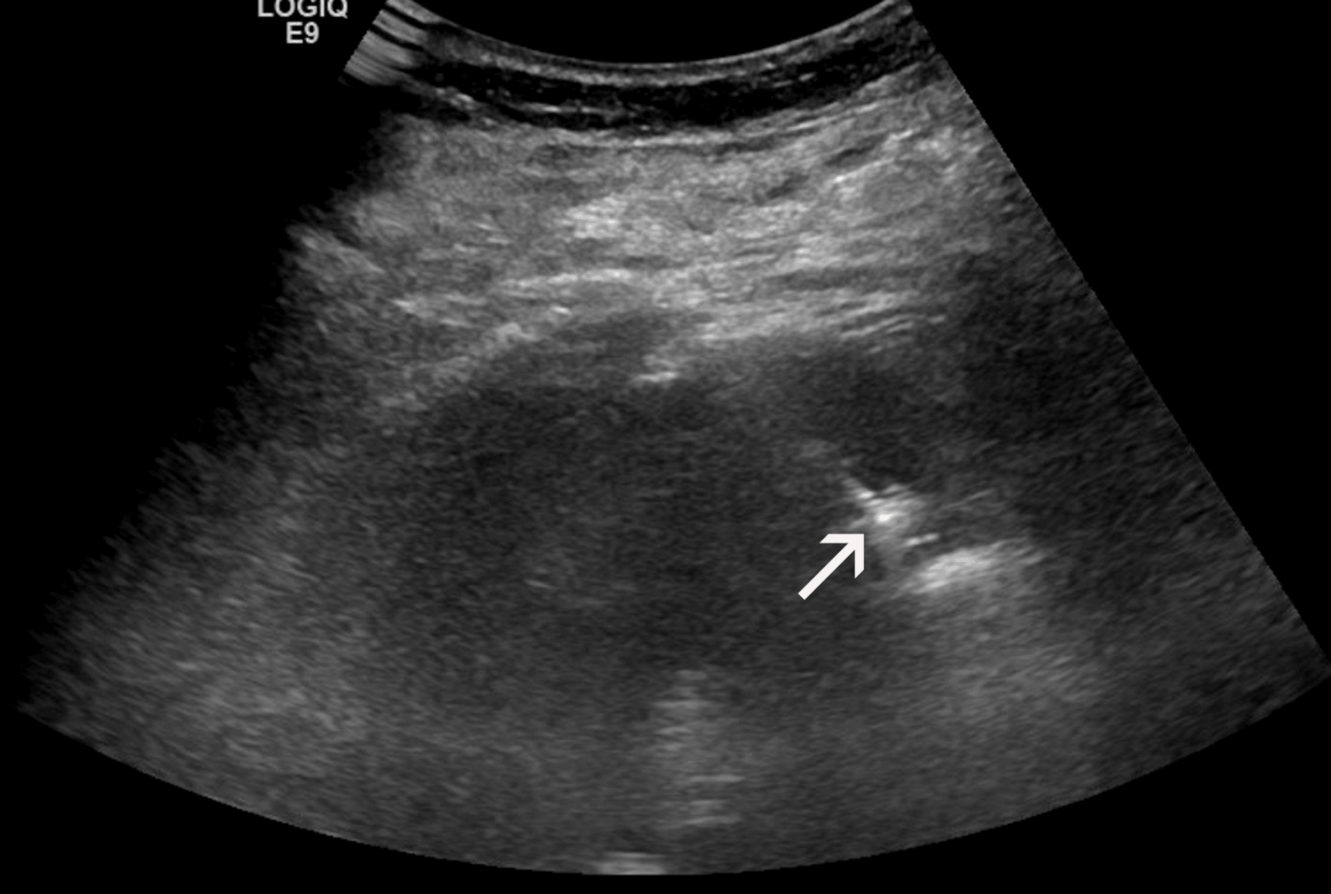
Coronal-oblique ultrasonographic image during microwave ablation (MWA). Image shows the tip of the MW antenna (arrow) at the deepest part of the tumor.

**Figure 3:**
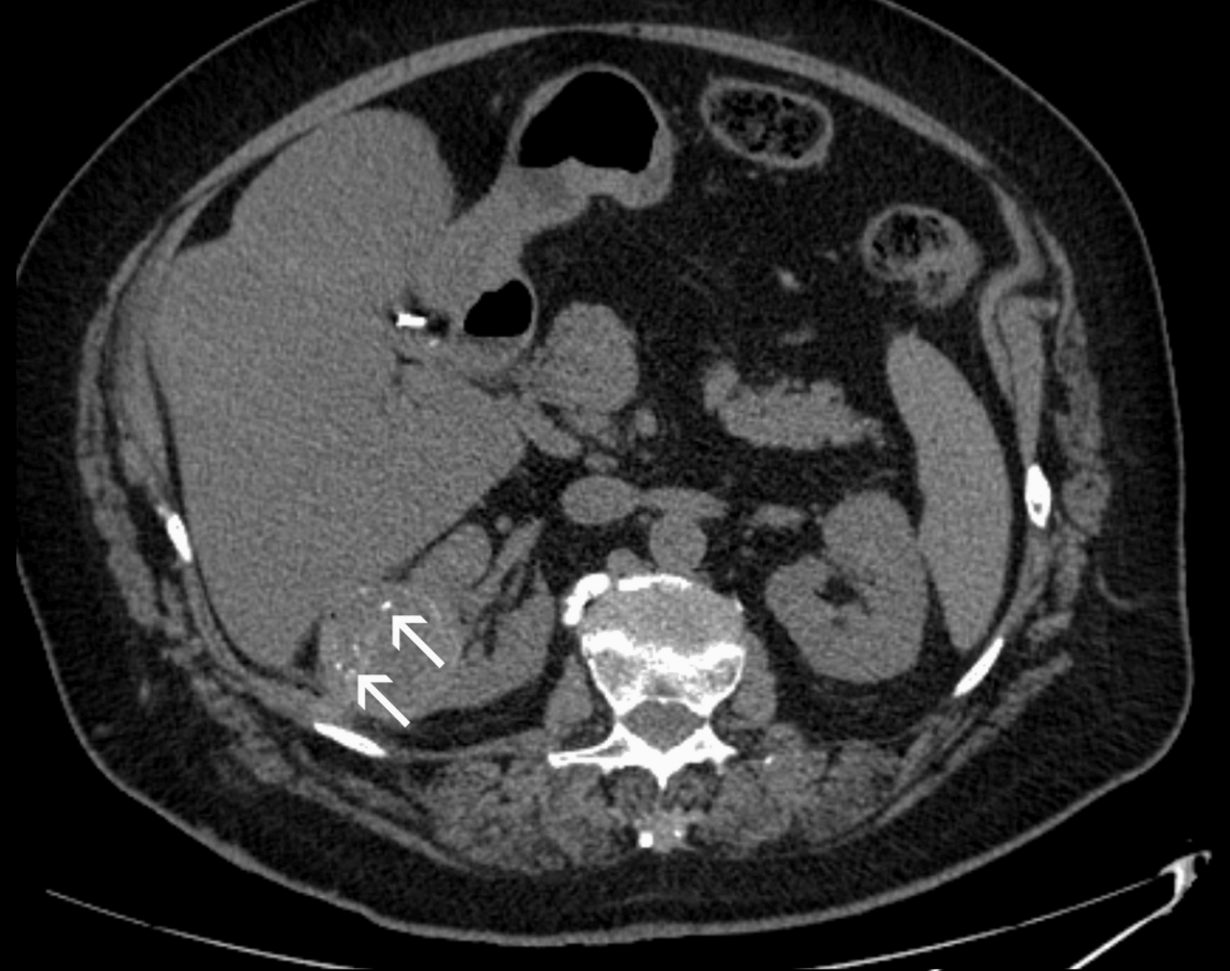
Unenhanced axial CT image immediately post ablation. No apparent complication following the procedure. Hyperdense intratumoral foci (arrows) are attributed to the embolic material (radiopaque microspheres) of TAE four weeks earlier.

Despite the absence of significant contraindications to surgery, the patient opted for IR management of her disease. She was informed in detail about the benefits, limitations, and potential complications of IR treatments and provided written consent. However, she refused to undergo a percutaneous biopsy prior to treatment. Taking into account the relatively large tumor size, transarterial embolization (TAE) with 70-150 μm radiopaque microspheres (DC-Bead Lumi, Boston Scientific, Marlborough, MA) of tumor feeders was first performed, to reduce tumor vascularity and to enhance the effect of subsequent ablation. The procedure was well-tolerated and uncomplicated. Microwave ablation (MWA) was performed four weeks post TAE. With the patient on conscious sedation and under ultrasonographic guidance, a 16-gauge microwave antenna with 18 mm active tip (ECO Medical Technology, Nanjing, China) was percutaneously placed in the inferior (caudal) part of the tumor with its tip at the tumor’s deepest border; ablation lasted eight minutes at 60 watts power. The antenna was subsequently withdrawn and reinserted at a position parallel to, and 2 cm cephalad to its first insertion, to treat the rest (superior part) of the tumor, with the same duration and power as the first ablation step (Figure 2). Track ablation was performed after each of the two ablation steps. Other than a mild, self-limiting hematuria, no immediate complications were observed (Figure 3), and the patient was discharged the following day. Laboratory tests post TAE and post MWA were unremarkable, and renal function remained normal.

**Figure 4:**
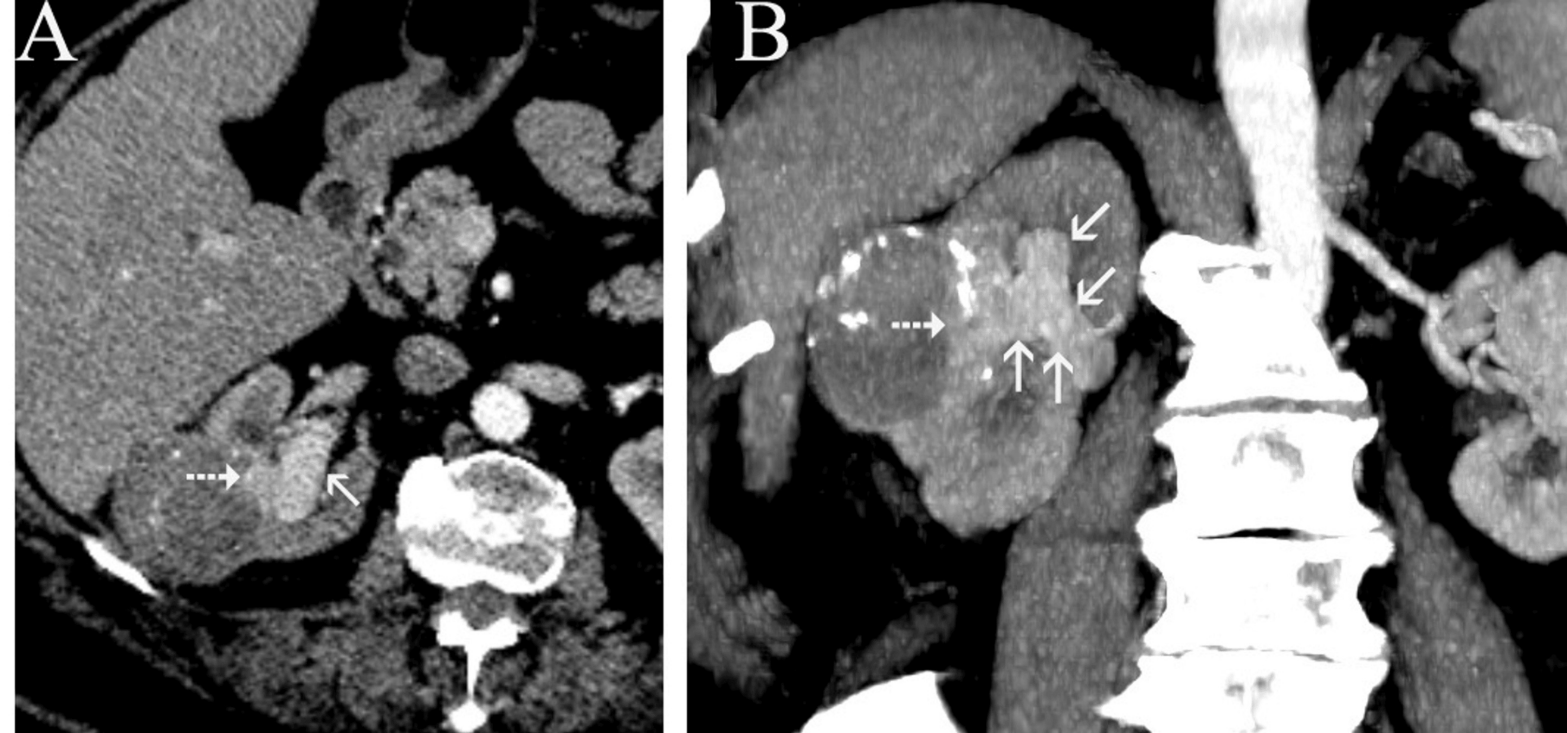
Contrast-enhanced, axial CT image (A) and coronal-oblique maximum intensity reconstruction (B), 10 weeks post ablation. Minimal residual enhancing tumor (dotted arrows in A and B), and marked involvement and dilatation of the pelvicalyceal system by enhancing tumor (arrows in A and B). Note the continuity and the similar enhancement between the residual and the newly appearing intrapelvicalyceal tumor.

**Figure 5:**
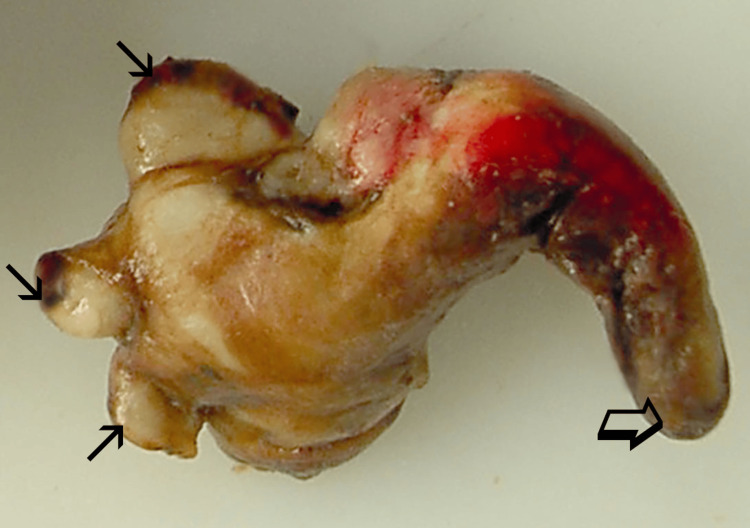
Pathologic image of the intrapelvicalyceal part of the tumor. The arrows indicate the protrusion of the tumor in renal calyces, and open arrow indicates the distal part of the tumor extending in the renal pelvis.

Ten weeks later, the patient presented with gross hematuria and right flank pain. CECT showed almost complete necrosis of the targeted tumor, but significant tumor extension in the upper calyces and in the renal pelvis with dilatation of them (Figures 4A-4B). There were still no other signs of local or distal tumor spread, and the patient's disease was characterized as T3a N0 M0 (stage 3). In light of these findings, open right nephroureterectomy was proposed (and accepted by the patient) as the only potentially curative treatment option. Macroscopic (Figure 5) and histopathologic examination of the surgical specimen confirmed extensive renal collecting system invasion by clear cell, grade II RCC in continuity with minimal viable tumor at the deepest part of the original lesion. Short-term follow-up (three months post-surgery) showed no significant complications or disease recurrence.

## Discussion

The first step (TAE) of the combined locoregional treatment of our case is an established interventional technique, which is often applied prior to ablation, to increase treatment efficacy and safety, particularly for relatively large (>3 cm) and centrally located renal tumors [4,5]. The herein presented complication is unrelated to TAE, as indicated by the immediate post-MWA CT scan, which showed no involvement of the pelvicalyceal system. On the other hand, it is highly likely that the invasion of the pelvicalyceal system was a consequence of the second part of the locoregional treatment (MWA), as indicated by the short time period between MWA and the detection of tumor spread into the pelvicalyceal system. A potential mechanism could be the inadvertent penetration of one of the upper renal calyces during the first or second placement of the MW antenna at the deepest part of the mass and subsequent implantation and growth of tumor cells in the pelvicalyceal system.

Urinary tract invasion by treatment-naive RCC has been occasionally reported in the literature as a sign of a locally advanced tumor. It has been theorized that RCC originating from the renal parenchyma invades the renal pelvis and/or calyces, and then fills in a "plastic" manner the low-resistance space of calyces, renal pelvis, and even ureter [6]. Cases of extensive filling of the ureter by RCC (even with tumor protruding from the ureter into the bladder) have been described [7]. Interestingly, ureteric wall invasion by the intraluminal part of the neoplasm is infrequent, and the latter receives its blood supply from the original "parenchymal" part of the RCC. Regarding the histologic subtype of the urinary tract-invading RCC, clear-cell seems to be the most common [6].

Percutaneous thermoablation of renal tumors may infrequently [8] cause complications involving the collecting system, such as ureteropelvic junction stenosis, urine leak, or obstruction due to blood clots [3,8]; however, to the best of the authors' knowledge, this is the first report of local RCC spread into the pelvicalyceal system, occurring as a consequence of percutaneous thermoablation. From a technical standpoint, it is difficult to predict or prevent this rare complication, particularly in tumors abutting the renal pelvis or calyces, because advancement of the electrode tip at the deepest part of the tumor is required to ensure complete ablation and an adequate safety margin. From a clinical standpoint, invasion of the pelvicalyceal system should be considered a major complication that worsens the prognosis of the RCC (increasing tumor stage from 1 to 3) and calls for radical surgical treatment.

Imaging differentiation between treatment-naive RCC invading the collecting system and urothelial carcinoma may be problematic. Homogeneous enhancement on CECT, hematuria, and an infiltrative growth pattern favor the diagnosis of urothelial carcinoma instead of an RCC invading the collecting system [9]. In the herein presented case, CT diagnosis was straightforward, owing to the continuity and the similar enhancement pattern between the newly appearing intra-pelvicalyceal part and the small residual part of the originally treated tumor.

This event also highlights the limitations of ablative therapies compared to more radical surgical treatments in terms of local tumour progression. Thorough post-procedural imaging and imaging follow-up should therefore be performed for the early diagnosis and management of such events.

## Conclusions

A rare and unexpected case of pelvicalyceal system temporally associated with invasion complicating locoregional treatment of RCC is presented. Pelvicalyceal system injury during the second step of the treatment (MWA), with subsequent implantation and growth of tumor cells in the pelvicalyceal system, is considered the most probable mechanism. The herein presented complication should be regarded as a major one and raise awareness when ablating tumors adjacent to the renal pelvis, since it increases the tumor stage and calls for radical surgical treatment.
